# Why so few Nobel Prizes for cancer researchers? An analysis of Nobel Prize nominations for German physicians with a focus on Ernst von Leyden and Karl Heinrich Bauer

**DOI:** 10.1007/s00432-021-03671-x

**Published:** 2021-05-29

**Authors:** Nils Hansson, Giacomo Padrini, Friedrich H. Moll, Thorsten Halling, Carsten Timmermann

**Affiliations:** 1 Department for the History, Philosophy, and Ethics of Medicine, Faculty of Medicine, Heinrich-Heine-University Duesseldorf, Moorenstr. 5, 40225 Düsseldorf, Germany; 2 Centre for the History of Science, Technology and Medicine, University of Manchester, Manchester, M13 9PL UK

**Keywords:** Excellence in cancer research, Nobel Prize, Ernst von Leyden, Karl Heinrich Bauer, Leonell Strong

## Abstract

**Purpose:**

To date, 11 scientists have received the Nobel Prize for discoveries directly related to cancer research. This article provides an overview of cancer researchers nominated for the Nobel Prize from 1901 to 1960 with a focus on Ernst von Leyden (1832–1910), the founder of this journal, and Karl Heinrich Bauer (1890–1978).

**Methods:**

We collected nominations and evaluations in the archive of the Nobel committee of physiology or medicine in Sweden to identify research trends and to analyse oncology in a Nobel Prize context.

**Results:**

We found a total of 54 nominations citing work on cancer as motivation for 11 candidates based in Germany from 1901 to 1953. In the 1930s, the US became the leading nation of cancer research in a Nobel context with nominees like Harvey Cushing (1869–1939) and George N. Papanicolaou (1883–1962).

**Discussion:**

The will of Alfred Nobel stipulates that Nobel laureates should have “conferred the greatest benefit to mankind”. Why were then so few cancer researchers recognized with the Nobel medal from 1901 to 1960? Our analysis of the Nobel dossiers points at multiple reasons: (1) Many of the proposed cancer researchers were surgeons, and surgery has a weak track record in a Nobel context; (2) several scholars were put forward for clinical work and not for basic research (historically, the Nobel committee has favoured basic researchers); (3) the scientists were usually not nominated for a single discovery, but rather for a wide range of different achievements.

## Introduction

Although cancer research has been a prevalent topic in the discussions among the Nobel Prize committee members since the first Nobel Prize in physiology or medicine was awarded in 1901, only eleven scientists have received the Nobel medal for work directly related to oncology. The list of Nobel laureates with cancer as a keyword in the official prize motivation includes scientists ranging from Johannes Fibiger in 1926 “for his discovery of the Spiroptera carcinoma” (Stolt et al. 2013) to James P. Allison and Tasuku Honjo in 2018 “for their discovery of cancer therapy by inhibition of negative immune regulation” (Table 1). Other prominent cancer researchers with a Nobel medal are Harald zur Hausen “for his discovery of human papilloma viruses causing cervical cancer” in 2008, J. Michael Bishop and Harold E. Varmus “for their discovery of the cellular origin of retroviral oncogenes” in 1989, David Baltimore, Renato Dulbecco and Howard Martin Temin “for their discoveries concerning the interaction between tumour viruses and the genetic material of the cell” in 1975, and the shared prize in 1966 between Peyton Rous “for his discovery of tumour-inducing viruses” and Charles Huggins “for his discoveries concerning hormonal treatment of prostatic cancer”.

**Table 1:** Laureates in physiology or medicine with “cancer” or “carcinoma” or “onco-” in the official prize motivation

Year	Laureates	Motivation
1926	Johannes Andreas Grib Fibiger	“for his discovery of the Spiroptera carcinoma”
1966	Peyton Rous Charles Brenton Huggins	“for his discovery of tumour-inducing viruses” “for his discoveries concerning hormonal treatment of prostatic cancer”
1975	David Baltimore, Renato Dulbecco, Howard Martin Temin	“for their discoveries concerning the interaction between tumour viruses and the genetic material of the cell”
1989	J. Michael Bishop, Harold E. Varmus	“for their discovery of the cellular origin of retroviral oncogenes”
2008	Harald zur Hausen	“for his discovery of human papilloma viruses causing cervical cancer”
2018	James P. Allison, Tasuku Honjo	“for their discovery of cancer therapy by inhibition of negative immune regulation”

In this article, we take a closer look at cancer researchers who were nominated for the award but never received it. The same approach has recently been adopted for other fields in medicine, e.g. in cardiovascular research (Drobietz et al 2021) and pharmacology (Pohar and Hansson 2020), to shed light on how physician-scientists were portrayed in this context. Going beyond the Nobel database (nobelprize.org), where nominees and nominators are listed from 1901 to 1953 with a brief (one sentence) description of the nomination, this article draws on full Nobel Prize nominations and evaluations by the Nobel committee to trace nominated cancer researchers. To date, only a small number of studies have looked at Nobel Prize links to cancer researchers (Kantha 2020). The researchers who have been subjects of such studies include the Tokyo pathologist Katsusaburo Yamagiwa (1863–1930), known for work on chemical carcinogenesis (Bartholomew 2002), the Heidelberg surgeon Vincenz Czerny (1842–1916) (Hansson and Tuffs 2016), nominated for new approaches to interdisciplinary cancer therapy, the German gynecologists Wilhelm Alexander Freund (1833–1917) and Bernhard Krönig (1863–1917) for surgical methods of treating uterus cancer (Hansson et al 2017), or the Berlin biochemist Carl Neuberg (1877–1956) for basic cancer research (Björk 2001). To that list, we can add nominees such as the immunologist and bacteriologist August von Wassermann (1866–1925), the pathologist Felix Marchand (1846–1928), immunologist and bacteriologist Paul Uhlenhuth (1870–1957), and the pathologist Robert Rössle (1876–1956), all proposed for cancer research (Table 2). Given that cancer research has been and is characterized by interdisciplinarity, many more researchers have been proposed for the award with links to the field. For example, the German zoologist and anatomist Theodor Boveri (1862–1915), who is best known for his ground-breaking work on cellular processes central to the etiology of cancer, was nominated in 1909, but for general work on cytology and the chemistry of fertilization. Therefore, he is not included in this overview.

**Table 2:** German scholars nominated for cancer research 1901–1953

Years	Candidate	Main motivation (related to cancer)	Nominations stating cancer	Total nominations
1915–1921	August von Wassermann	Artificial carcinoma in mice	3	45
1914–1917	Bernhard Krönig	Treatment of uterine cancer with X-Rays	4	4
1919	Carl Neuberg	Chemistry and therapy of cancer	1	11
1906	Ernst von Leyden	Cancer therapy	1	1
1923	Felix Marchand	Inflammation and tumors	1	9
1926–1931	Otto Warburg	Metabolism and characteristics of tumor cells	21	48
1907–1909	Paul Ehrlich	Cancer research	19	75
1926	Paul Uhlenhuth	Malignant tumors	1	40
1951	Robert Rössle	Tumorgenesis	1	3
1907	Vincenz Czerny	Research on cancer	1	3
1914	Wilhelm Freund	Extirpation of uterine cancer	1	2

In the following, our main focus is on two other scientists, who both had a lasting influence on the German scientific community identifying as cancer researchers: Ernst von Leyden (1832–1910) and Karl Heinrich Bauer (1890–1978).

## Ernst von Leyden and the Nobel Prize nomination in 1906

Ernst von Leyden grew up in Danzig (today Gdańsk, Poland) (His 1932). After his graduation at the “Medicinisch-chirurgisches Friedrich-Wilhelm-Institut” in Berlin, von Leyden began his career as a military physician in Berlin, then held positions at Düsseldorf and Königsberg, before in 1859 he returned to Berlin to become a senior physician under the direction of the internist Ludwig Traube (1818–1876). There he completed his habilitation thesis (Leyden 1863). After further work as a military physician, active also in the second Schleswig war (Schadewaldt 1985), in 1885 he was appointed director of the First Berlin Medical Clinic (1. Medizinische Klinik) of the Charité. At the Charité, he conducted research on various topics, including dietetics, neurology, tuberculosis and oncology. In tuberculosis research, he cooperated with his former student, otorhinolaryngologist Bernhard Fränkel (1836–1911) (Voswinckel 2019; Kuttner 1906; Rosenberg 1912), who later nominated him for the Nobel Prize (see below). At the turn of the twentieth century, Leyden published a number of articles on the etiology of cancer, which he suspected were caused by parasites. The means to cure cancer he proposed were similar to the ones he deemed suitable to fight tuberculosis, namely the institutionalization of cancer care and research with the establishment of sanatoria for patients suffering from cancer, especially as he did not believe a cure for cancer would be available in foreseeable time (Kohl 2016). To further strengthen the collaboration in Germany, von Leyden, in 1900, founded the “Comité für Krebssammelforschung”, with the goal to conduct statistical research on tumors. Notable members of this “Comité”, subsequently renamed to “Comité für Krebsforschung” were Paul Ehrlich (1854–1915) (Hüntelmann 2018), Vincenz Czerny and Bernhard Fränkel (Atzl and Helms 2012).

Using his extensive network in cancer and tuberculosis research, Leyden established Germany’s first cancer research institute in Berlin in 1903. One year later, jointly with Paul Ehrlich, he published the first issue of the International *Journal of Cancer Research and Clinical Oncology*, then called *Zeitschrift für Krebsforschung*, published in German until 1977 (Höffken 2004). Thus, Ernst von Leyden has played a key role in the institutionalization of cancer research in Germany.

It was Bernhard Fränkel who put him forward for the Nobel Prize in 1906. His nomination letter sent to Stockholm reflects the multifaceted work von Leyden had conducted during his career. Expressing the wish that “the honor of receiving the Nobel Prize should finally be granted to an internist” (Archive of the Nobel committee, yearbook 1906, Fränkel) he portrayed Ernst von Leyden as the only internist worthy of being awarded the Nobel Prize among German clinicians. Even though he was aware that von Leyden probably had not made a single, outstanding discovery, Fränkel argued that his lifetime achievements still made him prizeworthy because of his work on tuberculosis, asthma, and nutritional therapy. This “lifetime” argument mirrors the rhetoric in the Nobel nominations for Leyden’s colleague Vinzenz Czerny for efforts in interdisciplinary oncology (Hansson and Tuffs 2016). Regarding cancer research, Fränkel underlined that Leyden had “organized” the field. He further praised von Leyden for his commitment to teaching, and for coordinating the scientific society of internal medicine and its congresses. In the end, von Leyden did not make it onto the Nobel Committee’s shortlist in 1906, which in that year included Camillo Golgi (1843–1926), Santiago R. y Cajal (1852–1934), Jacques Loeb (1859–1924), Ernest Overton (1865–1933), August Bier (1861–1949), Carlos J. Finlay (1833–1915), Henry R. Carter (1852–1925), Alphonse Laveran (1845–1922) and Paul Ehrlich. The 1906 prize went to Golgi and Cajal “in recognition of their work on the structure of the nervous system.”

Even though Ernst von Leyden’s work did not convince the members of the Nobel Prize committee, he was undoubtedly a highly regarded scholar throughout his career. He was a corresponding member of the French Académie des Sciences and received multiple awards, among them the Cothenius Medal. A series of eponyms exist, including the Charcot-Leyden-Crystal, Leyden’s neuritis as well as Leyden-Möbius syndrome. These, naming discoveries related to asthma, neurology and the muscular system reflect his broad research interests. Furthermore, streets in several German cities bear his name, and a bust is displayed in front of the Charité medical clinic. Today, von Leyden is best remembered for his role in the institutionalization of cancer research in Germany. Thus, he is seen as an important part of the history of German medical societies such as the *German Society for Haematology and Medical Oncology*, with many publications and conventions in which von Leyden plays a major role (Voswinckel 2014). His legacy also lives on in the Ernst von Leyden Scholarship granted by the *Berlin Cancer Society* and the Ernst von Leyden Prize awarded by the *German Society for Gastroenterology, Digestive and Metabolic Diseases*.

## Karl Heinrich Bauer as Nobel candidate in the 1950s and 1960s

The name of K H Bauer is still well-known among German surgeons and cancer researchers (Lindner 1991; Kustrzycki et al. 2013). After studies in Erlangen, Heidelberg, Munich and Würzburg, he started his career as an unpaid junior doctor (“Volontärassistent”) at the pathological Institute of Freiburg under the guidance of Ludwig Aschoff (1866–1942). Bauer trained in surgery at the Georg-August University of Göttingen, where he completed his second academic qualification (Habilitation). Then, he worked at the University of Koenigsberg (today Kaliningrad) and with a grant from the Rockefeller Foundation he traveled around the Baltic Sea to Lithuania, Latvia, Estonia, Finland, Norway, Sweden, and Denmark. During this time, he published books about eugenics (Bauer 1926) and cancer (Bauer 1928). In 1933, Bauer succeeded the late Hermann Küttner (1870–1932) as director of the surgery department in Breslau (today: Wroclaw) where he continued his research, both in oncology (Bauer 1943, 1949a, b) and traumatology. In 1943, Bauer was appointed Head of Surgery at Heidelberg, where in August 1945, he was one of the professors credited with enabling the early re-opening of the university after the end of the Second World War. At Heidelberg his work increasingly exclusively focused on oncology: he published the best-known German textbook on cancer at the time (Bauer 1949a, b), and he was one of the driving forces for establishing the government-funded German Cancer Research Centre (Deutsches Krebsforschungszentrum, DKFZ) in Heidelberg (Hecker 1971). In the 1950s at the time of his first Nobel Prize nominations, he received several honors. He acted as president of two meetings of the German Surgical Association (1952 and 1958), and he chaired the *Naturforscherversammlung* at its 100th annual meeting in 1958. A medal with which the German Cancer Society (Deutsche Krebsgesellschaft) honours outstanding merits in cancer research was named after him.

### Nobel Prize nominations

In December 1953, Folke Henschen (1881–1977), professor of anatomical pathology at the Karolinska Institute 1920–1946, submitted a Nobel Prize nomination to the Nobel committee for physiology or medicine for the 1954 prize (Archive of the Nobel Committee, yearbook 1954, Henschen). Since the early 1930s, it had become common that the Nobel Prize in physiology or medicine was awarded jointly to two or three scientists and not only one, as earlier had been the rule, a fact that Henschen as a former member of the Nobel Committee was well aware of. Henschen put forward two candidates: Bauer and Leonell C. Strong (1894–1982, who in 1953 had left Yale University to become Director of the Biological Station at Roswell Park Memorial Institute, one of the leading cancer research institutions in the US). Henschen nominated the two for their research on the relation between cancer and heredity, more specifically for mutation processes in somatic cells. Underlining that Bauer had conducted research on cancer for 25 years, Henschen attached a chapter of Bauer’s book *Das Krebsproblem* to the nomination. He added that experimental research by Strong on the genetic links in the origin of cancer had provided “solid support” for Bauer’s theory and that it was of “fundamental importance”. As noted in Henschen’s autobiography (Henschen 1957), he had visited Strong in the US. Interestingly, Henschen mentioned that he himself “independently of Bauer and Strong” had made similar observations already in 1932, and he attached a manuscript from that year as evidence in support of this claim. We have seen in earlier studies on Nobel nominations that nominators sometimes used nominations of other researchers to draw attention to their own work (Hansson 2015). While his praise of Bauer was fairly general, Henschen was more specific about Strong’s achievements, which he summarized in four points:

1. The discovery that germinal changes comparable to mutations may be involved in the origin of cancer.
2. The determination that the characteristics of spontaneous transplantable and chemically induced tumors are influenced by intrinsic or constitutional factors derived from the ancestry of the host.
3. The observation that the methylcholanthrene, one of the powerful cancer-producing substances, can also induce mutations or general changes in mice. In fact methylcholanthrene is the best way of inducing mutations in mice.
4. The discovery that the age of the parent (birth rank or litter seriation) determines some of the characteristics of cancer in the offspring (mice).

Bauer and Strong were both nominated by Henschen, but even a quick look at their biographies and published work reveals that there were few commonalities. Bauer’s writings had their roots in constitutional pathology, an approach self-consciously positioned in opposition to bacteriology, experimental physiology, and other frameworks which the predominantly German promoters of constitutional medicine denounced as too reductionist and mechanistic, and ultimately un-German (Timmermann 2001). Strong’s can be located in an unapologetically reductionist tradition, that also may have had its roots in Germany, in the experimental physiology laboratories of Johannes Müller (1801–1858) and Carl Ludwig (1816–1895), and those who trained with them, and which came to provide the model for much biomedical research in the United States of America in the twentieth century (Fangerau 2010). Bauer’s *Das Krebsproblem* is an impressive textbook, but it sums up past findings and debates rather than pointing in new directions, and is distinctly German in style, at a time when this may have been viewed as increasingly old-fashioned. Bauer’s achievements in cancer research in the post-war period were predominantly administrative, leading to the establishment of the government-funded DKFZ in Heidelberg in 1964 (Wagner and Mauerberger 1989). Strong, in contrast, was a central pillar of the successful and very reductionist research programme around Clarence C. Little (1888–1971) and the creation of standardized mouse strains as disease models for laboratory-based cancer research (Löwy and Gaudilliére 1998).

Henschen’s nomination of Bauer and Strong led to a comprehensive special investigation by the Nobel committee, carried out by Karolinska Institute geneticist and cytologist Torbjörn Caspersson (1910–1997), head of the Institute of Medical Cell Research and Genetics, signed on 18 August 1954 (Archive of the Nobel Committee, yearbook 1954, Caspersson). At that time, Caspersson had recently published his landmark book *Cell growth and cell function: a cytochemical study* (1950). In his introduction of the 29 type-written pages, Caspersson noted that tumor research had experienced a boom during the last two decades [1930s and 40s]. Thus, according to Caspersson, the various methods and assumptions from a genetic perspective made such a Nobel evaluation of this vibrant field a meticulous task. He gave an overview on studies on tumor growth in mammals, fish and Drosophila, divided into (1) transplanted tumors, (2) spontaneous tumors and (3) induced tumors, citing work going forty years back. Focusing on Bauer, Caspersson noted that he had published a number of relatively general articles and books on cancer, along with more detailed articles on treatment methods and prophylaxis. “These works seem valuable for the treatments in medicine and surgery, but none of them contains such a significant discovery that it might be worthy of a Nobel Prize.” “In conclusion”, Caspersson added: “Bauer’s scholarly production covers a very wide spectrum in the tumor field. The focus of his original efforts is on treatment, first and foremost surgical treatment, but also chemotherapy. His efforts in the discussion about cancer prophylaxis is of great practical value […] but none of these aspects are prizeworthy.” Regarding Strong, however, the verdict was more positive: “If his works [as described by Henschen above] are verified in new experimental research, I find them to be of Nobel class, but for now he should not be considered for the prize” (Archive of the Nobel Committee, yearbook 1954, Caspersson).

During the Committee negotiations in the same year, the members judged the following scientists as “prize-worthy”: George Wells Beadle/Edward L. Tatum (genetics, Nobel Prize in physiology or medicine 1958), Georg von Békésy (research on the inner ear, Nobel Prize in physiology or medicine 1961), Charles H. Best (cholin functions), Frank MacFarlane Burnet/George Hirst (virus infections; Burnet was awarded the prize in physiology or medicine in 1960 with Peter Medawar), John Franklin Enders/Frederick O. Robbins/Thomas H. Weller (poliomyelitis, Nobel Prize in physiology or medicine 1954), Karl Folkers/Lester E. Smith (B 12), and Vincent du Vigneaud (structure of oxytocin, Nobel Prize in chemistry 1955). Finally, the 1954 physiology or medicine prize was awarded jointly to John Franklin Enders, Thomas Huckle Weller, and Frederick Chapman Robbins “for their discovery of the ability of poliomyelitis viruses to grow in cultures of various types of tissue.” In subsequent years, Bauer kept being nominated, e.g. in 1956 by the Erlangen otorhinolaryngologist Josef Beck (1891–1966), underlining research on “blood brain barrier”, but that did not lead to a new special investigation by the Nobel Committee. In 1961, Bauer was—along with urologist Charles Huggins—proposed anew, this time by the Frankfurt surgeon Rudolf Geissendörfer (1902–1976), who underlined publications around the “Mutationstheorie” (Bauer 1928). Geissendörfer, too, brought up the book *Das Krebsproblem*, “a one of a kind achievement […] one can say without exaggeration, that this book is a milestone for cancer research” (Archive of the Nobel Committee, yearbook 1961, Geissendörfer). During these years, cancer research was repeatedly on the agenda in the Nobel committee discussions. In 1959, for example, vice-chairman Axel Westman (1894–1960), gynecologist and obstetrician, voted for one half of the prize to Huggins and one half to George Papanicolaou (1883–1962), but the other committee members were in favor of Severo Ochoa and Arthur Kornberg who received it that year “for their discovery of the mechanisms in the biological synthesis of ribonucleic acid and deoxyribonucleic acid.” Nominations kept coming in for Huggins, who was awarded the prize seven years later, in 1966 (Hansson et al 2016).

## Discussion

The will of Alfred Nobel (written in 1895) stipulates that scientists should have “conferred the greatest benefit to mankind” to be eligible for the award. Cancer has been viewed as an insurmountable problem by many since the early Nobel Prize history, leading frequently into research cul-de-sacs. Leonell Strong, in a memoir published in 1976, quotes the German bacteriologist Paul Ehrlich, often celebrated as the father of chemotherapy: “Many a man has distinguished himself in science, then made a fool of himself by entering cancer research” (Strong 1976). Ehrlich himself had suggested that his ideas around magic bullets and ‘receptors’ (which we today think of as antibodies) might be applicable to cancer, but cancer chemotherapy only became a practical reality in the late 1940s and early 1950s, and immunotherapy more than two decades later again (Timmermann 2019). Cornelius Rhoads (1898–1959), one of the pioneers of cancer chemotherapy celebrated the first attempts with drugs targeted at cancer as posthumous success for Ehrlich on the centenary of the bacteriologist’s birthday in 1954. The centre of gravity in cancer research in the years after the Second World War was clearly shifting to the US, supported by astonishing amounts of research funding, from organisations such as the American Cancer Society, the government, culminating in President Nixon’s War on Cancer, and increasingly from pharmaceutical companies. While radical cancer surgery and radiotherapy had both European and American roots, cancer chemotherapy, immunotherapy and other more recent innovations in the treatment of cancer were developed mostly in the US, and had their conceptual origins in the laboratories of cell biologists and increasingly molecular biologists rather than in the clinic. This is mirrored by the Nobel Prizes awarded for cancer research since the 1950s.

As this article shows, a number of scientists were nominated as candidates for the Nobel Prize from 1901 to 1960 for innovations in the prevention or treatment of cancer. Why, then, was only Fibiger recognized with the Nobel medal during this period of time, a prize that in hindsight has been described as a Nobel error? Our analysis of the Nobel dossiers points at three main reasons.

First, many of the proposed cancer researchers were surgeons, and surgery has a weak track record in a Nobel context. During the first six decades of the twentieth century, only three prizes were awarded for the development of surgical procedures: Theodor Kocher (1841–1917) in 1909 ‘for his work on the physiology, pathology and surgery of the thyroid gland’, Alexis Carrel (1873–1944) in 1912 ‘in recognition of his work on vascular suture and the transplantation of blood vessels and organs’, António Egas Moniz (1874–1955) in 1949 ‘for his discovery of the therapeutic value of leucotomy in certain psychoses’. In the pool of nominees in surgery, the Nobel committee was more interested in brain, heart, and transplant surgery than surgeons specializing on cancer operations (Hansson et al 2019).

Second, several scholars were put forward for clinical work and not for basic research, whereas the Nobel committee over time has favoured basic researchers. This mirrors the fact that most of the Nobel committee members were basic scientists.

Third, as the nomination letters for Leyden and Bauer exemplify, several scientists were nominated for contributions in a wide range of different research, or as “doyen” in the scientific community. The mission of the Nobel Committee, however, was and is to pinpoint single, groundbreaking discoveries and attribute them to between one and three scientists. In the second half of the twentieth century, more prizes were awarded for cancer research, partly explained by the rise of genetics. Further research aims at analyzing the shift from a “Nobel centre” during the first decades until the US overtook this position with prominent nominees like Harvey Cushing (1869–1939) (brain tumors) and George N. Papanicolaou (Pap smear). On a more general level, we argue that Nobel Prize nominations provide valuable information to reconstruct research trends and networks in cancer research.

## Data Availability

The datasets generated and analysed during the current study are available from the corresponding author on reasonable request.
